# Collision-Induced Gas-Phase Reactions of PFB-TMS Derivatives of F_2_-Prostaglandins in Quadrupole GC-NICI-MS/MS: A Mini-Review and a Meta-Analysis

**DOI:** 10.3390/molecules30193846

**Published:** 2025-09-23

**Authors:** Dimitrios S. Tsikas, Stefanos A. Tsikas

**Affiliations:** 1 Core Unit Proteomics, Institute of Toxicology, Hannover Medical School, 30623 Hannover, Germany; 2 Dean of Studies Office—Academic Controlling, Hannover Medical School, 30623 Hannover, Germany

**Keywords:** eicosanoids, fragmentation, ionization, mass spectrometry, mechanisms, pathways, PCA, ROCA

## Abstract

Arachidonic acid (eicosatetraenoic acid) is the precursor of the eicosanoids, which include prostaglandins (PG). Methods based on GC-MS/MS are the Gold Standard for the quantitative analysis of eicosanoids in biological samples. After extraction and derivatization, biological F_2_-prostaglandins are analyzed on quadrupole GC-MS/MS apparatus as pentafluorobenzyl (PFB) ester trimethylsilyl (TMS) ether derivatives, i.e., PFB-TMS. Negative-ion chemical ionization (NICI) in the ion source generates abundant anions due to [M-PFB]^−^, which are detected in the selected ion monitoring (SIM) mode. Collision-induced dissociation (CID) of [M-PFB]^−^ in the collision cell generates numerous product ions, which are suitable candidates for quantitative analyses in the selected reaction monitoring (SRM) mode. In this article, we report on investigations of gas-phase reactions of PFB-TMS derivatives of F_2_-prostaglandins, which consist of PGF_2α_, 8-*iso*-PGF_2α_, and up to 62 further isomers, known as the F_2_-isoprostanes. We performed a meta-analysis of previously reported CID mass spectra (32 eV) of PFB-(TMS)_3_ of seven chemically closely related isomeric F_2_-prostaglandins of the 15-F_2t_-IsoP type. This unique dataset contains 19 product ions generated by CID of the common precursor at *m*/*z* 569 [M-PFB]^−^ in the *m*/*z* range of 150–600. All isomers produced the same product ions, which, however, greatly differed in their intensity. Principal Component Analysis (PCA) and Receiver Operating Characteristic (ROC) Analysis (ROCA) were performed. Two compounds, i.e., 8-*iso*-9β,11α-PGF_2α_ and 9α,11β-PGF_2α_, and two product ions, i.e., *m*/*z* 299 [M-PFB-3×TMSOH]^−^ and *m*/*z* 215 [M-PFB-3×TMSOH-C_4_H_8_-C_2_H_4_]^−^, were noticeable. ROCA revealed the highest disagreement between PGF_2α_ and 8-*iso*-9β,11α-PGF_2α_ (AUC = 0.7075 ± 0.0834, *p* = 0.0248). PCA and ROCA are of limited value in the GC-MS/MS of closely chemically related F_2_-prostaglandins. Fragmentation mechanisms were proposed for the formation of all 19 product ions generated by CID of common precursor anions due to [M-PFB]^−^.

## 1. Introduction

**Figure 1:**
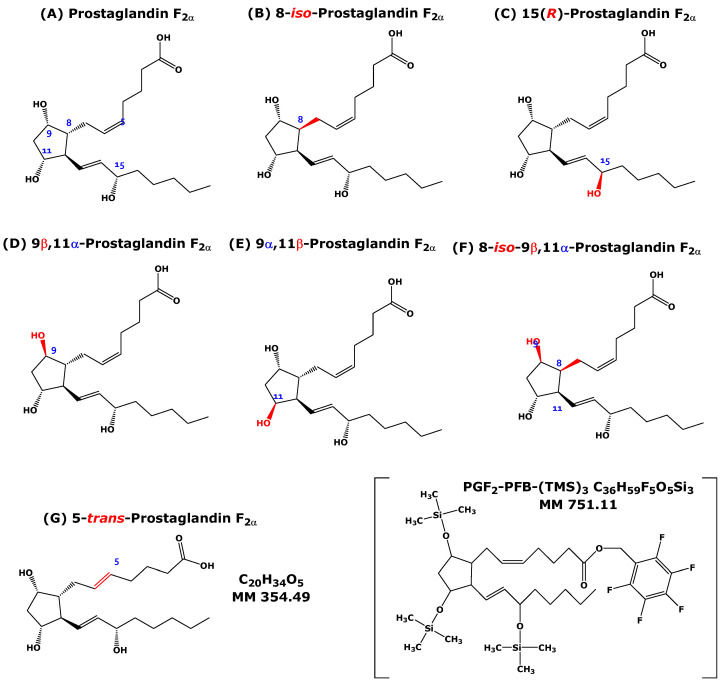
Chemical structures of the F_2_-prostaglandin isomers (MM, 354.49) investigated by Ferretti and Flanagan [4]. Red-colored parts indicate the specific isomer with respect to prostaglandin F_2α_. The framed structure on the bottom shows the pentafluorobenzyl (PFB) ester trimethylsilyl (TMS) ether (PFB-TMS) derivative of prostaglandin F_2_ without indication of the stereochemistry. The molecular mass (MM) of the PFB-TMS derivative of all F_2_-prostaglandin isomers amounts to 751.

Arachidonic acid, i.e., eicosatetraenoic acid, is the precursor of multiple metabolites, collectively named eicosanoids [1,2]. They include prostaglandins (PGs) and leukotrienes (LTs). Cyclooxygenase (COX) catalyzes the formation of prostaglandin (PG) PGF_2α_ ((5*Z*,9α,11α,13*E*,15*S*)-9,11,15-trihydroxyprosta-5,13-dien-1-acid) and can also catalyze the formation of its isomer 8-i*so*-PGF**_2α_** ((5Z,9α,11α,13*E*,15*S*)-9,11,15-trihydroxyprosta-5,13-dien-1-acid). Four classes of F_2_-prostaglandins, widely known as F_2_-isoprostanes, differ in regiochemistry (Figure S1). In theory, 64 isomers of F_2_-prostaglandins may exist in total [3]. Besides PGF**_2α_** and 8-*iso*-PGF_2α_, F_2_-prostaglandins include the structures shown in Figure 1 (see also Table S1 in the Supplementary Materials). They all have the formula C_20_H_34_O_5_, the molecular mass 354.48, a cyclopentane ring, and three hydroxyl groups at C-9, C-11, and C-15. Two OH groups are positioned on the cyclopentane ring (C-9 and C-11). The cyclopentane ring carries two residues on C-8 and C-12, which are differently oriented in space. The **α**-chain on C-8 carries a carboxylic group, while the **β**-chain or *w*-chain on C-12 is a hydroxylated olefinic alkyl. These F_2_-prostaglandin isomers have been analyzed by GC-MS/MS in the NICI mode by Ferretti and Flanagan [4]. The GC-NICI-MS/MS mass spectra of these isomeric F_2_-prostaglandins are the subject of the present work.

**Figure 2:**
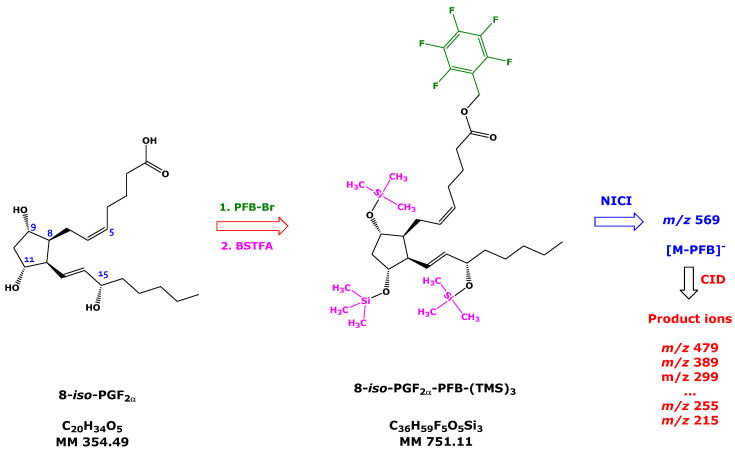
Derivatization of 8-*iso*-PGF_2α_ representing the F_2_-isoprostanes for GC-NICI-MS or GC-NICI-MS/MS analysis. Derivatization of 8-*iso*-PGF_2α_ first with PFB-Br and then with BSTA generates the PFB-TMS derivative of 8-*iso*-PGF_2α_ (8-*iso*-PGF_2α_-PFB-(TMS)_3_). NICI of this derivative forms the most abundant anion at *m*/*z* 569 [M-PFB]^−^. Collision-induced dissociation (CID) of [M-PFB]^−^ generates numerous product ions, including *m*/*z* 489, 389, 299, 255, and 215. BSTFA, *N*,*O*-bis(trimethylsilyl)trifluoroacetamide; NICI, negative-ion chemical ionization; PFB, pentafluorobenzyl; PFB-Br, pentafluorobenzyl bromide; TMS, trimethylsilyl. See also Figures S2 and S3.

F_2_-Isoprostanes have been analyzed in biological samples by GC-MS or GC-MS/MS as pentafluorobenzyl (PFB) ester trimethylsilyl (TMS) ether derivatives (PFB-TMS) in the NICI mode (Figure 2). In the first derivatization reaction, the carboxylic group is converted by PFB-Br in acetonitrile to its PFB ester derivative. This reaction is catalyzed by organic bases such as *N*,*N*-diisopropylethylamine. Subsequently, the PFB ester derivative is silylated by means of silylating agents such as BSTFA (*N*,*O*-bis(trimethylsilyl)trifluoroacetamide) to generate the PFB ester TMS ether derivatives [5]. Divergence in urinary 8-*iso*-PGF**_2α_** concentrations from GC-NICI-MS/MS quantification after thin-layer chromatography and immunoaffinity column chromatography revealed the heterogeneity of 8-*iso*-PGF**_2α_**, which consists at least of 15(*S*)-8-*iso*-PGF**_2α_** [5] and presumably of 15(*R*)-8-*iso*-PGF**_2α_** [6]. Non-derivatized F_2_-isoprostanes and other eicosanoids can be analyzed by LC-MS/MS using negative electrospray ionization (NESI), i.e., LC-NESI-MS/MS [2,6].

Quantitative determination of eicosanoids by GC-NICI-MS is performed by selected ion monitoring (SIM) of [M-PFB]^−^ ions that are generated by NICI of PFB-TMS derivatives (Figure 2). In the case of F_2_-prostaglandins, the common anions with *m*/*z* 569 [M-PFB]^−^ are generated by NICI of the PFB-TMS derivatives of all F_2_-prostaglandin isomers. The number of isobaric F_2_-prostaglandins is high. Anions with *m*/*z* 569 [M-PFB]^−^ are the most intense and therefore the most useful ions in quantitative GC-NICI-MS analyses of F_2_-prostaglandins by SIM. The concentration of F_2_-prostaglandins in biological samples is very low, usually lying in the pM range [5,6]. For these reasons and because of coelution or incomplete GC separation, GC-NICI-MS methods lack specificity for particular F_2_-prostaglandins.

In general, higher specificity of eicosanoid analysis is achieved by quantitative GC-NICI-MS/MS analysis in the selected reaction monitoring (SRM) mode, even in the case of coelution of derivatives. Given the high chemical similarity of the F_2_-prostaglandins, selection of both specific and abundant product ions generated by CID of the precursor ion *m*/*z* 569 [M-PFB]^−^ is not trivial (Figure S2), but requires detailed GC-NICI-MS/MS studies with available synthetic F_2_-prostaglandin compounds.

In this article, we review CID gas-phase reactions of PFB-TMS derivatives of F_2_-prostaglandins, which consist of PGF_2α_, 8-*iso*-PGF**_2α_,** and up to 62 further isomers, known as the F_2_-isoprostanes. The paper is a meta-analysis of the CID mass spectra (32 eV) of the PFB-(TMS)_3_ derivatives of seven chemically closely related isomeric F_2_-prostaglandins that have been previously reported by Ferretti and Flanagan [4] (Figure 1). This unique dataset contains 19 product ions generated by CID of the common precursor ions at *m*/*z* 569 [M-PFB]^−^ in the *m*/*z*-range 150–600. Fragmentation pathways are proposed for the formation of the 19 product ions of the seven F_2_-prostaglandins shown in Figure 1.

Principal Component Analysis (PCA) and Receiver Operating Characteristic (ROC) Analysis (ROCA) are widely used chemometric analytical tools in qualitative studies to detect and visualize differences and agreements between tested groups of analytes, notably in metabolomics studies [7,8,9,10,11]. In the present study, we tested the utility of PCA and ROCA as additional analytical approaches to resolve the CID behavior of the seven isomeric F_2_-prostaglandins shown in Figure 1.

## 2. Methods

Ferretti and Flanagan performed a GC-NICI-MS/MS study on synthetic, structurally closely related F_2_-prostaglandins of the 15-F_2t_-IsoP type, including PGF_2α_ and 8-*iso*-PGF_2α_ [4]. 8-*iso*-PGF_2α_ is one of the best investigated F_2_-isoprostanes [3]. Ferretti and Flanagan generated GC-NICI-MS/MS mass spectra (32 eV) of seven F_2_-prostaglandins (Figure 1), analyzed the individual GC-NICI-MS/MS mass spectra, and tabulated the *m*/*z* and relative intensity values of the remaining, non-fragmented precursor ion with *m*/*z* 569 and of the 19 product ions in the *m*/*z* range of 150–600. Unfortunately, retention times had been reported only for the PFB-TMS derivatives of 8-*iso*-PGF_2α_ (9.4 min) and of 9α,11β-PGF_2α_ (10.9 min). In the present work, we used the originally reported data [4] to reconstruct the GC-NICI-MS/MS mass spectra (see Figure S3 and Table 1 below). We conducted PCA, ROCA, and other statistical tests to visualize and detect potential differences between the seven F_2_-prostaglandins. Such analyses could be useful to interpret the GC-NICI-MS/MS mass spectra and to outline potential CID mechanisms that may depend upon the structure of the chemically closely related F_2_-prostaglandins. In this context, data previously reported by our group on stable-isotope-labeled 8-*iso*-PGF_2α_ and its metabolites were used to complement the analyses [12,13].

**Figure 3:**
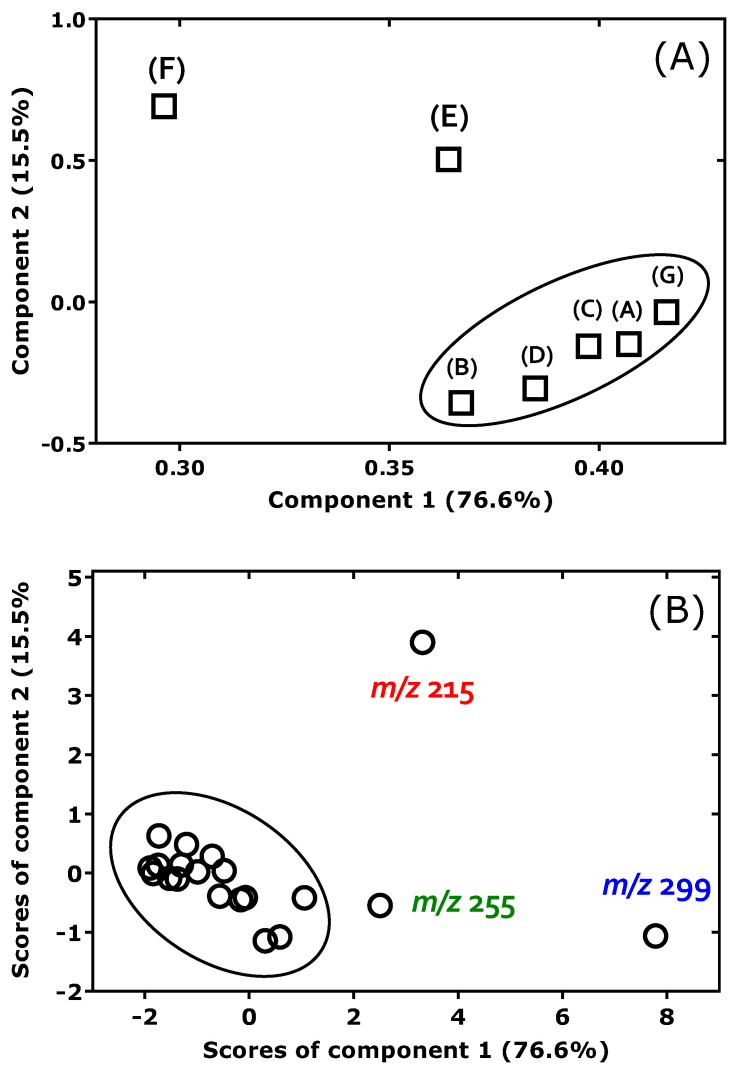
Principal Component Analysis (PCA). (**A**) The loading plot visualizes the contribution of each PGF_2_ isomer to the first two principal components, which together cover the dominant patterns of variance (92% explained) across the dataset. Each point represents a compound’s loading on Component 1 (*x*-axis, Eigenvalue = 5.36) and Component 2 (*y*-axis, Eigenvalue = 1.08). (**B**) The PCA score plot illustrates the distribution of the 19 product ions in the space defined by the first two principal components. Each point represents the position of each product ion along Component 1 and Component 2. The data reported by Ferretti and Flanagan [4] were used (see Table 1).

PCA was performed with STATA 14 (StataCorp, College Station, TX, USA) and with SIMCA 13.0.2 (UMETRICS AB, Umea, Sweden) in unit variance (UV) scaling. Data were further analyzed with PLS-DA, OPLS, and OPLS-DA. The validity of the obtained models was assessed using the cross-validation parameters (R2X: R2Y and Q2Y), in combination with loadings, permutation, and VIP plots, and the *p*-value of cross-validated analysis of variance (CV-ANOVA). Only features with VIP value > 1 were considered statistically significant. GraphPad Prism was used for statistical analyses and preparation of graphs. PCA was conducted on the relative intensity values of the product ions of the seven F_2_-prostaglandins obtained from the GC-NICI-MS/MS analyses originally performed by Ferretti and Flanagan [4]. A plot of compound loadings and component scores retrieved from PCA is graphically presented in Figure 3.

GraphPad Prism Version 7 for Windows (GraphPad Software, San Diego, CA, USA) and STATA 14 (StataCorp, College Station, TX, USA) were used for PCA, k-means cluster analysis, and bi- and multivariate statistical tests. ROCA was used to calculate area under the curve (AUC) values and evaluate agreement/disagreement between the seven F_2_-prostaglandins. The Wilcoxon matched-pairs signed-rank test was used in two-tailed paired analyses. A *p*-value of <0.05 was considered significant.

Chemical structures and names of the investigated native and derivatized F_2_-prostaglandins, as well as of product ions, were drawn and suggested by ChemDraw 15.0 Professional (PerkinElmer, Rodgau, Germany). The Clean Up Structure tool was used in drawing the structures of F_2_-prostaglandins.

## 3. Results

Twenty product ions, including the remaining, non-fragmented precursor ion *m*/*z* 569 (intensity range, 7–42%), were detected within a relative intensity ranging from <1% to 100%. The same product ions were observed from all F_2_-prostaglandins. F_2_-Prostaglandins with product ions with intensity values <1% had generally intense product ions of very low intensity. The most intense product ions (base peaks, intensity of 100%) were *m*/*z* 299 for the F_2_-prostaglandins, abbreviated as A, B, C, D, and G, and *m*/*z* 215 for the F_2_-prostaglandins, abbreviated as E and F (Table 1).

The results of PCA, based on the data listed in Table 1, are presented in Figure 3. Figure 3A shows that all F_2_-prostaglandins load positively on Component 1 (Eigenvalue 5.36; 76.6% of the total variance explained), suggesting that this component reflects a shared variance structure across compounds, likely associated with overall intensity or common chemical behavior. In contrast, Component 2 (Eigenvalue 1.08; 15.5% of the total variance explained) differentiates the variables more distinctly: the isomers 8-*iso*-9β,11α-PGF_2α_ and PGF_2α_ have strong positive loadings, while the remaining five isomers exhibit negative loadings of varying magnitude. This separation implies that Component 2 covers a secondary dimension of variation, potentially related to structural isomerism or specific fragmentation behavior in the collision cell of the GC-MS/MS apparatus. F_2_-Prostaglandins clustering closely in the plot, e.g., PGF_2α_ and 15(*R*)-PGF_2α_, indicate closely related MS/MS properties. Thus, PGF_2α_ and 15(*R*)-PGF_2α_ differ “only” in the spatial orientation of the OH group on C-15 on the β-chain (i.e., *S* versus *R*).

The PCA score plot in Figure 3B illustrates the distribution of the product ions from *m*/*z* 569 in the space defined by the first two principal components, which together explain 92% of the variance in the data. Each point represents the position of each product ion along Component 1 and Component 2. The score plot reveals considerable spread along Component 1, suggesting that the *x*-axis covers the dominant variance across the *m*/*z* values, likely driven by overall intensity differences of PGF_2_ isomers with high loadings on Component 1 as seen in the loading plot (Figure 3A). Component 2 contributes less to the overall separation but highlights a small number of *m*/*z* values with distinctive profiles, notably *m*/*z* 215 (strongly positive on Component 2) and *m*/*z* 299 (strongly negative on Component 2) (Figure 3B).

Guided by visual inspection of the PCA score plot, *k*-means clustering (*k* = 3) was applied, resulting in three chemically distinct sample groups (“cluster”):

(1) Group 3: *m*/*z* 299 and *m*/*z* 215 (i.e., the two outliers in Figure 3B).
(2) Group 2: *m*/*z* 569, *m*/*z* 317, *m*/*z* 273, *m*/*z* 255, *m*/*z* 219, and *m*/*z* 161.
(3) Group 1: all remaining *m*/*z* values.

The location of Groups 3 and 2 at the periphery of the PCA plot (Figure 3B), especially along Component 2, supports their outlier-like behavior in terms of multivariate compound intensities.

Statistical testing confirmed that the clusters differ significantly: a Kruskal–Wallis test indicated group differences in the intensity values of individual PGF_2_ isomers, and a MANOVA showed a jointly significant multivariate difference across the PGF_2_ isomers. These results suggest that PCA-based clustering illustrates differences between groups with regard to their CID behavior.

**Figure 4:**
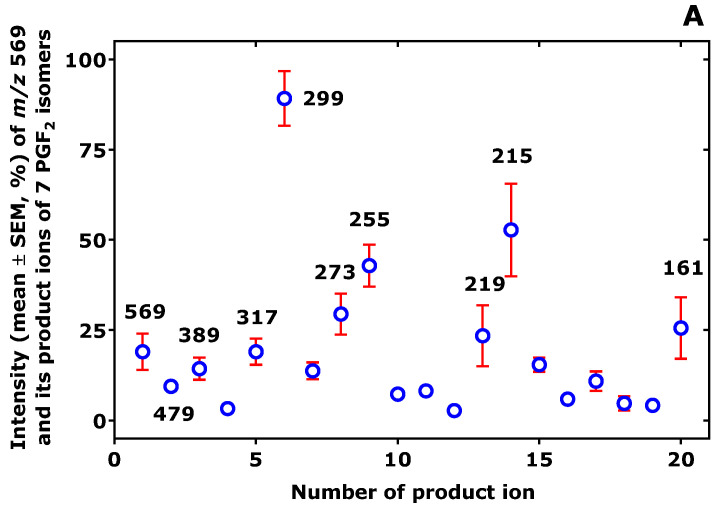

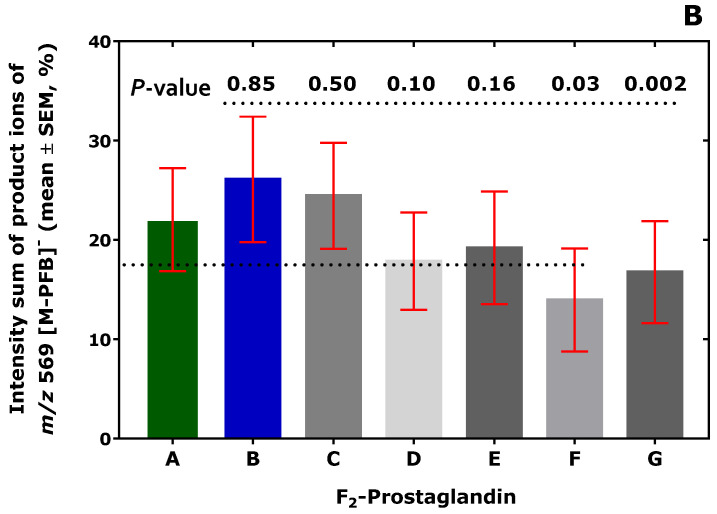
Statistical analysis of the data summarized in Table 1. (**A**) Mean intensity with SEM as error bars of the precursor *m*/*z* 569 and of the indicated product ion of the seven PGF_2_ isomers investigated. (**B**) Mean intensity sum with SEM as error bars of the product ions of the precursor *m*/*z* 569 for the indicated PGF_2_ isomers. The intensity values of the indicated isomers were compared with those of PGF_2α_ by a paired two-sided Wilcoxon test. The insert above the columns in (**B**) shows the *p*-values of this comparison. The data reported by Ferretti and Flanagan [4] were used (see Table 1).

Figure 4 shows the results of the statistical analysis of the intensity values of the ions summarized in Table 1. The most intense ions were found to be *m*/*z* 299, 215, 255, 273, 161, and 219.

No correlations were found between 8-*iso*-9β,11α-PGF_2α_ and PGF_2α_, 8-*iso*-PGF_2α_, 15(*R*)-PGF_2α_, 9β,11α-PGF2α, or 5-*trans*-PGF_2α_ (see Supplementary Materials). The highest Spearman correlation coefficient (*r*_S_) was observed between 8-*iso*-PGF_2α_ and 15(*R*)-PGF_2α_: *r*_S_ = 0.903 and *p* = 5×10^−8^. It should be noted that urinary 8-*iso*-PGF_2α_ consists of 15(*S*)-8-*iso*-PGF_2α_ and 15(*R*)-8-*iso*-PGF_2α_ [5,6]. In the present case, no data were available for 15(*R*)-8-*iso*-PGF_2α_ in the study reported by Ferretti and Flanagan [4]. We have to assume that 8-*iso*-PGF_2α_ analyzed by Ferretti and Flanagan [4] is identical to 15(*S*)-8-*iso*-PGF_2α_.

ROCA of the data revealed AUC values for the ROC curves, which were statistically significantly different only for the comparison of PGF_2α_ with 8-*iso*-9β,11α-PGF_2α_: AUC = 0.7075 ± 0.0834, *p* = 0.0248 (mean ± SEM) (see Supplementary Materials).

Commercially available [3,3,4,4-^2^H_4_]-8-*iso*-PGF_2α_ has been used as an internal standard for the quantitative determination of 8-*iso*-PGF_2α_ in biological samples, including urine and plasma or serum, by GC-NICI-MS and GC-NICI-MS/MS. [3,3,4,4-^2^H_4_]-8-*iso*-PGF_2α_ (i.e., 15(*S*)-[3,3,4,4-^2^H_4_]-8-*iso*-PGF_2α_) has also been used to study the NICI and CID of their PFB-TMS derivatives. In general, the use of ^2^H labels is useful in mechanistic studies, as they provide additional information that is not revealed by using unlabeled material such as 8-*iso*-PGF_2α_.

The most intense ions in the GC-NICI-MS mass spectra were at *m*/*z* 569, 573, and 573 due to [M-PFB]^−^. No loss of ^2^H from [3,3,4,4-^2^H_4_]-8-*iso*-PGF_2α_ was observed, indicating that NICI did not influence the stability of the labels in the PFB-(TMS)_3_ derivatives under the conditions used.

Table 2 summarizes the most intense product ions in the GC-NICI-MS/MS mass spectra of the PFB-(TMS)_3_ derivatives of 8-*iso*-PGF_2α_ and [3,3,4,4-^2^H_4_]-8-*iso*-PGF_2α_ (i.e., both 15(*S*)-8-*iso*-PGF_2α_ and 15(*S*)-[3,3,4,4-^2^H_4_]-8-*iso*-PGF_2α_), and of PGF_2α_ and [3,3,4,4-^2^H_4_]-PGF_2α_. Neutral loss of one TMSOH group (90 Da) led to [M-PFB-1×TMSOH]^−^ at *m*/*z* 479, 483, and 483, respectively, with an intensity of about 15% each. Neutral loss of one (CH_3_)_2_Si=CH_2_ group (72 Da) generated [M-PFB-1×TMSOH]^−^ at *m*/*z* 407, 411, and 411, respectively, with an intensity of about 12% each. The positions of the TMSO groups that lost TMSOH and (CH_3_)_2_Si=CH_2_ are unknown. Several isomeric anions are likely to co-exist.

2,3-Dinor-8-*iso*-PGF_2α_ and 2,3-dinor-5,6-dihydro-8-*iso*-PGF_2α_ are enzymatic metabolites of 8-*iso*-PGF_2α_ (Figure S4), and *ent*-2,3-dinor-5,6-dihydro-8-*iso*-PGF_2α_ is considered a metabolite of *ent*-8-*iso*-PGF_2α_. These isoprostanes have been analyzed by GC-NICI-MS and GC-NICI-MS/MS as PFB-(TMS)_3_ derivatives [13].

Table 3 summarizes the most intense ions in the GC-NICI-MS and GC-NICI-MS/MS mass spectra of the PFB-(TMS)_3_ derivatives of synthetic 2,3-dinor-5,6-dihydro-8-*iso*-PGF_2α_, *ent*-2,3-dinor-5,6-dihydro-8-*iso*-PGF_2α_. The α-chain of the 8-*iso*-PGF_2α_metabolites is by two CH_2_ groups shorter than its precursor in 2,3-dinor-8-*iso*-PGF_2α_; in 2,3-dinor-5,6-dihydro-8-*iso*-PGF_2α_, the C-5=C-6 double bond is saturated (Figure S4).

**Table 3:** Mass fragments (*m*/*z*, intensity, %) in the GC-NICI-MS and GC-NICI-MS/MS mass spectra of the PFB-TMS derivatives of 2,3-dinor-5,6-dihydro-8-*iso*-PGF_2α_ and *ent*-2,3-dinor-5,6-dihydro-8-*iso*-PGF_2α_. The ions [M-PFB]^−^ (P, precursor) were subjected to CID with argon (0.15 Pa) with a collision energy of 25 eV. NICI with methane (65 Pa) was performed (electron current, 200 eV; electron current, 600 µA). The instrument TSQ 7000 was used. The table was constructed with data reported in Ref. [13].

Ion Assignment	2,3-Dinor-5,6-dihydro-8-*iso*-PGF_2α_	*ent*-2,3-Dinor-5,6-dihydro-8-*iso*-PGF_2α_
GC-NICI-MS		
[M-PFB]^−^	543 (100)	543 (100)
[M-PFB-TMSOH]^−^	453 (4)	453 (3)
[M-PFB-TMSOH-(CH_3_)_2_Si=CH_2_]^−^	381 (6)	381 (5)
GC-NICI-MS/MS		
[P]^−^	543 (8)	543 (10)
[P-TMSOH]^−^	453 (5)	453 (4)
[P-2×TMSOH]^−^	363 (4)	363 (5)
[P-2×TMSOH-(CH_3_)_2_Si=CH_2_]^−^	291 (12)	291 (14)
[P-3×TMSOH]^−^	273 (100)	273 (100)
see Figure 5	247 (22)	247 (21)
see Figure 5	229 (35)	229 (31)

**Figure 5:**
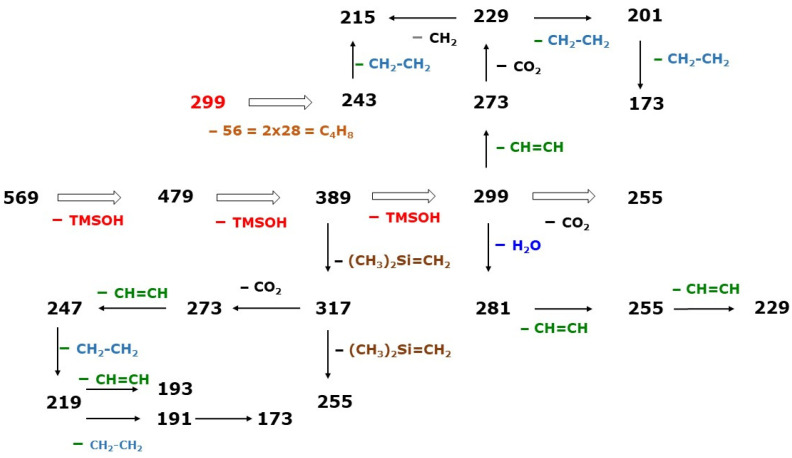
Proposed fragmentation pathways for the formation of the indicated product ions produced by CID of the ion *m*/*z* 569 [M-PFB]^−^ generated by NICI of the PFB-TMS derivatives of prostaglandin F_2_ isomers, investigated by Ferretti and Flanagan [4]. See also Table 1.

The most intense ions in the GC-NICI-MS mass spectra were at *m*/*z* 543 due to [M-PFB]^−^. The most intense product ions in the GC-NICI-MS/MS mass spectra generated from the precursor ion at *m*/*z* 543 were *m*/*z* 273 due to [M-PFB-3×TMSOH]^−^.

## 4. Discussion

### 4.1. Mechanistic Aspects of CID Fragmentations of [M-PFB]^−^ Ions of PFB-TMS Derivatives

In laboratory mass spectrometers, including GC-MS and GC-MS/MS, such as quadrupole instruments, gas-phase reactions can be investigated [14,15,16]. Under NICI conditions, PFB-TMS derivatives of analytes such as the F_2_-prostaglandins ionize to form mainly anions due to [M-PFB]^−^ by neutral loss of PFB radicals (Figure S2). The negative charge in these precursor anions is located on the carboxylate O atoms. CID of precursor ions with *m*/*z* 569 [M-PFB]^−^ from PFB-(TMS)_3_ derivatives of isomeric F_2_-prostaglandins, such as 8-*iso*-PGF_2α_ (Figure 2), generates several products as a result of collisions of accelerated precursor ions with argon atoms in the collision cell [14,15,16]. Studies by Ferretti and Flanagan [4] revealed that CID of *m*/*z* 569 of seven chemically closely related isomeric F_2_-prostaglandins (Figure 1) generated 19 product ions each, yet with greatly differing intensity values (Table 1). In the present article, we performed a meta-analysis of this unique dataset from various points of view.

Characteristic GC-NICI-MS/MS product ions of [M-PFB]^−^ precursor ions of PFB-TMS derivatives of F_2_-prostaglandins are ions due to neutral loss of trimethylsilanol (TMSOH, 90 Da) groups. CID of the common precursor *m*/*z* 569 [M-PFB]^−^ of the PFB-TMS of the PGF_2_ isomers generates product ions with *m*/*z* 479 [M-PFB-1×TMSOH]^−^, *m*/*z* 389 [M-PFB-2×TMSOH]^−^, and *m*/*z* 299 [M-PFB-3×TMSOH]^−^, obviously by consecutive loss of three TMSOH groups from the three etherified OH groups of the F_2_-prostaglandins. The negative charge of these product ions is most likely located on the carboxylate O atoms. Each TMSOH group is generated from the TMSO ether moiety and from one H atom of the two neighboring CH_2_ groups. Its neutral loss/elimination leads to the formation of a -C=C-bond. For example, loss of the TMSO group on C-15 on the β-chain of 8-*iso*-PGF_2α_ may form the C-14=C-15 bond as well as the C-15=C-16 bond. Thus, the product ion with *m*/*z* 299 is likely to be a trienoic carboxylic acid that may consist of up to six isomers. It is possible that the structures surrounding the TMSO-carrying C atoms, such as the cyclopentane ring or an existing -C=C-bond, may play a role in the elimination of TMSOH groups. All possible structures of the isomeric ion with *m*/*z* 299 contribute collectively to the detected signal generated by the product ion *m*/*z* 299.

NICI of PFB-TMS derivatives of eicosanoids is also associated with neutral losses of TMSOH groups, yet at a much lower extent compared to the CID of [M-PFB]^−^ (see Table 2). Obviously, neutral losses of TMSOH groups require higher energy than that provided by the precursor ions in the form of translational collision energy. That can be set by the experimenter, for instance, to 32 eV as carried out by Ferretti and Flanagan [4].

It is notable that electron ionization (EI) of PFB-TMS derivatives is associated with cleavage (fragmentation of the carbon backbone) of C-C bonds on the right (α1) and/or on the left (α2) of the C atom that carries a TMSO ether moiety rather than with elimination of TMSOH groups, especially in PFB-TMS derivatives of unsaturated hydroxylated fatty acids such as leukotriene B_4_ and its metabolites [17].

**Scheme 1:**
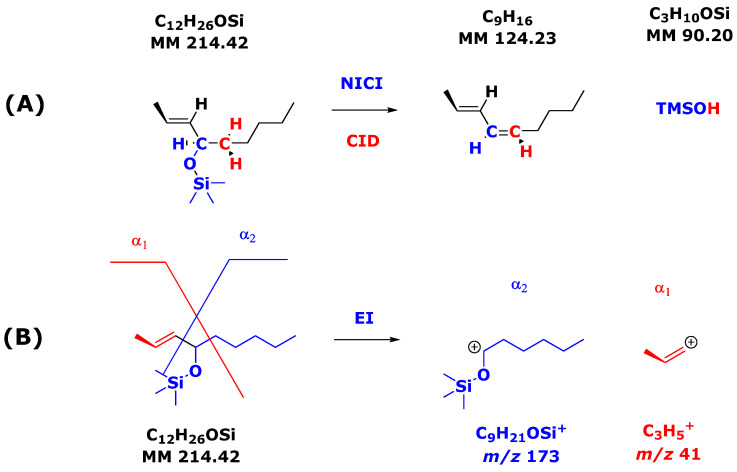
Comparison of (**A**) negative-ion chemical ionization (NICI) and collision-induced dissociation (CID) with (**B**) electron ionization (EI) of a hypothetical molecule as a trimethylsilyl derivative. EI includes an α-cleavage that may result in two complementary ions α_1_ and α_2_. For more detail, see the text.

The differences between the NICI and EI of PFB-TMS derivatives are likely to be based on the ionization energy that prevails in the ion-source chamber, for instance, 70 eV in EI versus << 70 eV in NICI. Under CID conditions, the collision energy may exceed by far the electron energy under NICI, such as in the case of F_2_-prostaglandins, so that C-C-cleavages may occur in addition to the elimination of TMSOH groups as shown in Scheme 1 below.

From the electronic perspective, the loss of a TMSO group from a CH group, e.g., CH-CH-OTMS, corresponds to a one-electron reduction in the C atom that carries the TMSO group from the oxidation number ±0 to −1. The loss of one H atom from the vicinal CH_2_ or CH group corresponds to a one-electron oxidation of the neighbor C atom from the oxidation number −3 to −2. Thus, the CID neutral loss/elimination of TMSOH groups and the accompanying formation of C=C-bonds is a one-electron intramolecular redox reaction from C^±0^/C^−3^ to C^−1^/C^−2^. The neutral loss/elimination of TMSOH and the formation of C=C-double bonds in eicosanoids have been described as a remote-site mechanism [17]. It takes place without the appearance of a new charge or the disappearance of an existing electric charge.

The information contained in Table 1 was analyzed with respect to neutral losses in addition to the 90 Da neutral loss of TMSOH. Multiple differences between the *m*/*z* values of the product ions of *m*/*z* 569 [M-PFB]^−^ of the PFB-TMS derivatives of the PGF_2_ isomers investigated by Ferretti and Flanagan [4] were found and assigned as follows:

5 times for 90 Da due to TMSOH;
2 times for 72 Da due to (CH_3_)_2_Si=CH_2_;
3 times of 44 Da due to CO_2_;
5 times for 28 Da due to H_2_C=CH_2_;
6 times for 26 Da due to HC≡CH;
6 times for 18 Da due to H_2_O.

The numbers above suggest that neutral losses may have occurred in many different ways, which do not correspond to the number of the three hydroxyl groups, the two -C=C- double bonds, and the single carboxylic group of each PGF_2_ isomer. Obviously, the CID process is very complex; the large number of product ions resembles EI mass spectra. One may assume that innumerable argon atoms collide (in the collision cell) with each atom and/or functional groups of the accelerated precursor ions with *m*/*z* 569 [M-PFB]^−^ and possibly with certain intermediates and product ions. One may imagine that CID activates the entity of the molecules of the precursor ions, which means that their structural differences could vanish to a great extent and generate very similar CID mass spectra.

Precursor ions of 8-*iso*-PGF_2a_ and its metabolites labeled with ^18^O exclusively in the carboxylic groups were found to lose TMS^18^OH, which is strong evidence for a transfer of a TMS from an OTMS group to the carboxylate O atom to form an ^18^OTMS ester and to finally lose TMS^18^OH [12,13]. This CID reaction has been named a Murphy-type rearrangement [18]. This rearrangement can occur once only, and the position of the thioether moiety in the precursor ion is unknown, but could, in part, contribute to the differences in CID mass spectra of the PFB-TMS derivatives of the F_2_-prostaglandin isomers. The sequence of the neutral loss of the three TMSOH groups is unknown.

**Figure 6:**
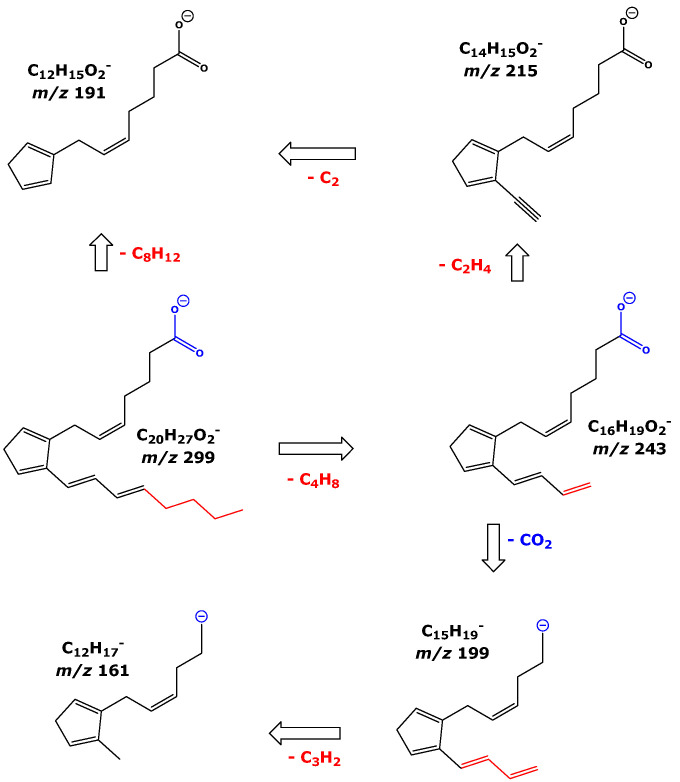
Proposed fragmentation pathways and chemical structures of the product ions with *m*/*z* 161, 191, 199, 215, and 243, presumably deriving from the product ion with *m*/*z* 299 produced by CID of the ions [M-PFB]^−^ with *m*/*z* 569 generated by NICI of the PFB-TMS derivatives of prostaglandin F_2_ isomers, investigated by Ferretti and Flanagan [4]. Red- and red-colored parts indicate the fragments that were left from preceding decomposed ions. See also Table 1.

Figure 6 shows proposed mechanisms for the formation of the product ions *m*/*z* 243, 215, 199, 191, and 161 that may have been derived from *m*/*z* 299. Because of their relatively small *m*/*z* values, the product ions with *m*/*z* 161, 191, 199, 215, and 243 are likely to form by step-wise decomposition of the β-chain of the F_2_-prostaglandins (see Figure S4 for the donor metabolites). The product ions with *m*/*z* 199 and 161 are carbanions, presumably resulting from the loss of their carboxylic groups as CO_2_. The product ions *m*/*z* 161, 191, 199, 215, 243, and 299 are likely to contain a cyclopentadiene ring.

**Scheme 2:**
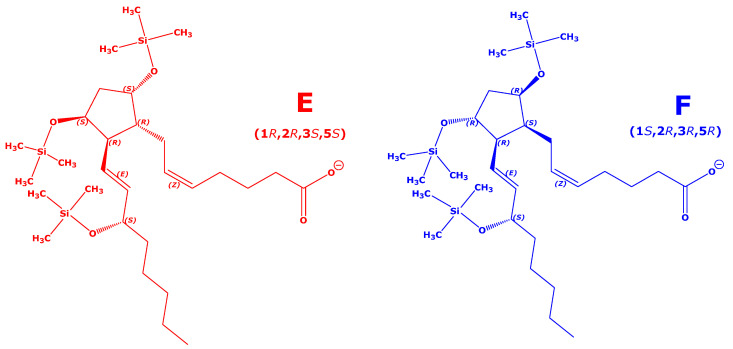
Chemical structures of the F_2_-prostaglanin isomers (E) and (F) with a high extent of disagreement with respect to their stereochemistry. Constructed with the ChemDraw software by using the tool *Stereochemistry*. For more detail, see the text.

Figure 6 schematically summarizes the proposed fragmentation steps, including the neutral losses for the formation of the indicated product ions. They were produced by CID of the precursor ion *m*/*z* 569 [M-PFB]^−^ that was generated by NICI of the PFB-TMS derivatives of the prostaglandin F_2_ isomers investigated by Ferretti and Flanagan [4]. The product ion with *m*/*z* 299 [M-PFB-3×TMSOH]^−^ is likely to undergo four different fragmentation mechanisms. One of these mechanisms leads to the formation of *m*/*z* 215, which is the most intense product ion of 9α,11β-PGF_2α_ (E) and 8-*iso*-9β,11α-PGF_2α_ (F). It should be noted that (E) and (F) have a high extent of disagreement with respect to their stereochemistry, i.e., they behave like enantiomers with a mirror image except for the β-chain (see Scheme 2). This feature may have facilitated the formation of *m*/*z* 215 as the most abundant product ion.

### 4.2. PCA, ROCA

Usually, PCA and ROCA in GC-MS, LC-MS [6,7,8,9,10,11], and other analytical techniques are widely performed on datasets that include several groups of analytes, treatments including drugs, diseased and healthy subjects that usually serve as a control, or in animals. In the present study, we performed PCA and ROCA on only seven structurally highly similar F_2_-prostaglandins. The dataset consisted of 20 common *m*/*z* values, i.e., product ions including the common precursor ion *m*/*z* 569 [M-PFB]^−^ of seven F_2_-prostaglandins, and their intensity values as a single variable. Unfortunately, retention times of only two F_2_-prostaglandins were reportedly available. Also, the ion intensity values stemming from single analyses should be noted, i.e., we have no knowledge of their variance. We acknowledge that these are limitations of our study with respect to the PCA and ROCA.

Nevertheless, the GC-MS/MS dataset from the study by Ferretti and Flanagan [4], which we meta-analyzed in the present work, is unique in the literature on F_2_-prostaglandins [3]. Both PCA and ROCA revealed two isomers, i.e., 8-*iso*-9β,11α-PGF_2α_ (compound E) and 9α,11β-PGF_2α_ (compound F), and two product ions, i.e., *m*/*z* 299 [M-PFB-3×TMSOH]^−^ and *m*/*z* 215 [M-PFB-3×TMSOH-C_4_H_8_-C_2_H_4_]^−^, which were noticeable. In the current analytical case with a small dataset and only one variable, the districting features revealed by PCA and ROCA were observable to the naked eye, especially when the data are listed in a clear table. Our study suggests that PCA and ROCA may be of limited usefulness in the very specific case of GC-NICI-MS/MS analysis of PFB-TMS derivatives of closely related F_2_-prostaglandins.

## 5. Conclusions

The eicosanoids are a large family of arachidonic acid-derived organic carboxyl groups containing endogenous molecules. In theory, solely the primary F_2_-prostaglandins amount to up to 64. Reliable quantitative analysis of biological F_2_-prostaglandins is highly challenging, even for GC-NICI-MS/MS. NICI of PFB-TMS derivatives of F_2_-prostaglandins generates the common ion M-PFB]^−^ with *m*/*z* 569. CID of the precursor carboxylate ions generates many product ions, which are common to all F_2_-prostaglandins but may differ in their intensity. We meta-analyzed the data reported by Ferretti and Flanagan [4] on the GC-NICI-MS/MS analysis of the PFB-(TMS)_3_ derivatives of seven chemically closely related F_2_-prostaglandin isomers of the 15-F_2t_-IsoP type, including PGF_2α_ and 8-*iso*-PGF_2α_. A meta-analysis of data previously reported on ^2^H-labeled 8-*iso*-PGF_2α_ and its dinor-dihydro metabolite complemented this analysis. By means of these datasets, the 32-eV CID behavior and the formation of 19 product ions from [M-PFB]^−^ of the seven F_2_-prostaglandin isomers was elucidated. Identified neutral losses included TMSOH (90 Da), CH_3_)_2_Si=CH_2_ (72 Da), CO_2_ (44 Da), H_2_C=CH_2_ (28 Da), HC≡CH (26 Da), and H_2_O (18 Da). PCA and ROCA of the GC-NICI-MS/MS mass spectra identified 8-*iso*-9β,11α-PGF_2α_ and 9α,11β-PGF_2α_ as the isomers with the greatest disagreement. Based on these data, CID fragmentation mechanisms were proposed. Neutral losses of TMSOH are prominent intramolecular redox-based elimination reactions that lead to the formation of C=C-bonds within the cyclopentane ring and in the β-chain of F_2_-prostaglandin isomers of the 15-F_2t_-IsoP type.

Although unique in the literature, the utility of the data meta-analyzed here is of limited value to delineate, in depth, the CID reactions of PFB-TMS derivatives of closely related F_2_-prostaglandin isomers. Additional experimental, for instance, use of different unlabeled and stable-isotope-labeled precursors, collision energy, and argon pressure values for other eicosanoids [18,19], and theoretical studies, such as density functional theory (DFT) computations reported on structurally unrelated molecules [20,21], are warranted for F_2_-prostaglandin isomers.

## Figures and Tables

**Table 1:** Product ions (left column) generated by CID of the precursor (P) ions [M-PFB]^−^, which were generated by NICI of the PFB-TMS derivatives of the seven F_2_-prostaglandins. The numbers in the other columns are the relative intensity values of the product ions. The table was constructed with data from Ferretti and Flanagan [4]. The chemical structures of the F_2_-prostaglandins are shown in Figure 1.

Product Ions (*m*/*z*)	(A) PGF_2a_	(B) 8-*iso*-PGF_2α_	(C) 15(*R*)-PGF_2α_	(D) 9β,11α-PGF_2α_	(E) 9α,11β-PGF_2α_	(F) 8-*iso*-9β,11α-PGF_2α_	(G) 5-*trans*-PGF_2α_
569 (P)	13	42	34	12	13	7	12
479	5	7	9	12	14	15	4
389	24	14	24	16	10	<1	11
363	8	7	<1	<1	<1	<1	4
317	27	24	33	18	13	6	12
299	100	100	100	100	75	49	100
281	18	13	24	6	13	15	7
273	12	42	52	26	23	13	38
255	55	68	30	48	40	23	36
247	13	8	10	7	3	3	7
243	10	7	10	9	7	8	6
229	4	3	1	3	3	<1	4
219	24	70	26	16	9	<1	18
215	41	31	44	12	100	100	41
201	17	15	10	23	20	8	15
193	11	4	7	8	3	<1	7
191	9	8	22	12	18	<1	6
177	4	<1	4	<1	6	16	<1
173	3	<1	<1	13	6	4	<1
161	43	57	47	14	7	6	5

**Table 2:** Major mass fragments (*m*/*z*, intensity, %) in the GC-NICI-MS/MS mass spectra of the PFB-TMS derivatives of 8-*iso*-PGF_2α_, PGF_2α_, and their tetradeuterated analogs. The ions [M-PFB]^−^ (P, precursor) were subjected to CID with argon (0.2 Pa) with a collision energy of 18 eV. NICI with methane (65 Pa) was performed (electron current, 90 eV; electron current, 220 µA). The instrument TSQ 45 was used. The table was constructed with data reported in Ref. [12].

8-*iso*-PGF_2α_	^2^H_4_-8-*iso*-PGF_2α_	PGF_2α_	^2^H_4_-PGF_2α_	Ion Assignment
569 (26)	573 (15)	569 (10)	573 (17)	[P]^−^
479 (17)	483 (15)	479/12)	483 (30)	[P-1×TMSOH]^−^
389 (32)	393 (37)	389 (32)	393 (400)	[P-2×TMSOH]^−^
317 (23)	321 (33)	317 (46)	321 (28)	P-2×TMSOH-(CH_3_)_2_Si=CH_2_]^−^
299 (100)	303 (100)	299 (100)	303 (100)	[P-3×TMSOH]^−^
273 (33)	277 (60)	273 (50)	277 (37)	[P-2×TMSOH-(CH_3_)_2_Si=CH_2_-CO_2_]^−^
255 (76)	259 (62)	255 (62)	259 (33)	[P-3×TMSOH-CO_2_]^−^

## Data Availability

No new data were created or analyzed in this study. Data sharing is not applicable to this article.

## Supplementary Materials

The following supporting information can be downloaded at https://www.mdpi.com/article/10.3390/molecules30193846/s1. Figure S1. Chemical structures and names of the four series (types) of F_2_-isoprostanes. Table S1. Names and abbreviations of the F_2_-prostaglandin investigated by Ferretti & Flanagan [4]. Figure S2. Simplified schematic of the GC-NICI-MS/MS analyses performed in individual experiments by Ferretti and Flanagan [4] on seven F_2_-prostaglandins (A, B, C, D, E, F, and G) as their PFB-TMS derivatives. NICI in the ion source using methane as the reagent gas resulted in the formation of several ions (A-x^−^… G-x^−^; A-2x^−^… G-2x^−^). The most abundant ions were *m*/*z* 569 due to [M-PFB]^−^, i.e., A^−^, B^−^, C^−^, D^−^, E^−^, F^−^, and G^−^; ^−^ symbolizes the negatively charged carboxylate group. These ions were selected by quadrupole Q1 and forwarded to the collision cell, the quadrupole Q2. Collision-induced dissociation (CID) of the precursor ions *m*/*z* 569 with argon atoms generated product ions due to elimination of TMSOH groups (90 Da) and CO_2_ (44 Da) as neutral losses and decomposition of the backbone (A-x^−^… G-x^−^; A-2x^−^… G-2x^−^; A-ω^−^… G-ω^−^). The precursor ions and 19 product ions were selected by scanning the quadrupole Q3 (A-x^−^… G-x^−^; A-2x^−^… G-2x^−^; A-ω-1^−^… G-ω-1^−^) to generate the GC-NICI-MS/MS mass spectra. The collision energy was 32 eV for all F_2_-prostaglandins [4]. Figure S3. Product ions generated by CID of the precursor ions [M-PFB]^−^ with *m*/*z* 569 formed by NICI of the PFB-TMS derivatives of the prostaglandin F_2_ isomers, investigated in the study. The GC-NICI-MS/MS mass spectra were reconstructed by GraphPad Prism using the data reported by Ferretti and Flanagan [4]. Figure S4. Chemical structures of 8-*iso*-PGF_2α_, 2,3-dinor-8-*iso*-PGF_2α_, and 2,3-dinor-5,6-dihydro-8-*iso*-PGF_2α_.
