# Supplementary material for: Collision-Induced Gas-Phase Reactions of PFB-TMS Derivatives of F2-Prostaglandins in Quadrupole GC-NICI-MS/MS: A Mini-Review and a Meta-Analysis
Source: Molecules. 2025 Sep 23;30(19):3846. doi: 10.3390/molecules30193846 (PMC12526261; doi:10.3390/molecules30193846)
Supplement: Supplementary file 1 [file molecules-30-03846-s001.zip › molecules-3837455-SI.pdf]

molecules

MDPI

Review

Collision-Induced Gas-Phase Reactions of PFB-TMS Derivatives of F_2-P s in Quadrupole GC-NICI-MS/MS: A Mini-Review and a Meta-Analysis

Dimitrios S. Tsikas [1,*] and Stefanos A. Tsikas [2]

[1] Core Unit Proteomics, Institute of Toxicology, Hannover Medical School, 30623 Hannover, Germany
[2] Dean of Studies Office—Academic Controlling, Hannover Medical School, 30623 Hannover, Germany; tsikas.stefanos@mh-hannover.de
* Correspondence: tsikas.dimitros@mh-hannover.de

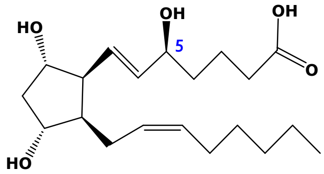

5*S*,9α,11α-trihydroxy-(8β)-prosta-6*E*,14*Z*-dien-1-oic acid

5-F_{2t}-IsoP

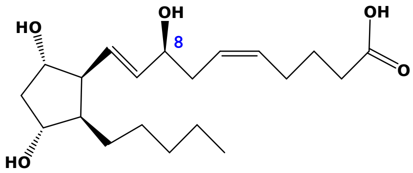

5*S*,9α,11α-trihydroxy-2,3,5-trinor-1,1,1-trihomo-18,19,20-trinor-(8β)-prosta-2*Z*,6*E*-dien-1-oic acid

8-F_{2t}-IsoP

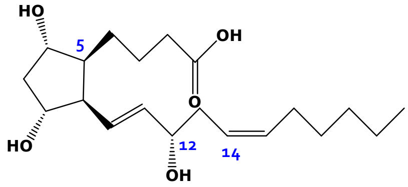

9α,11α,15*S*-trihydroxy-2,3,5-trinor-20,20,20-trihomo-(8β)-prosta-13*E*,17*Z*-dien-1-oic acid

12-F_{2t}-IsoP

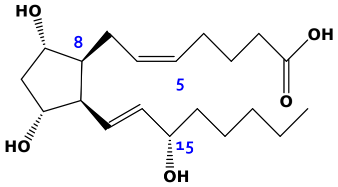

9α,11α,15*S*-trihydroxy-(8β)-prosta-5*Z*,13*E*-dien-1-oic acid

8-*iso*-$PGF_{2\alpha}$, 15-F_{2t}-IsoP

Figure S1. **Chemical structures and names of the four series (types) of F_2-isoprostanes.**

Besides $PGF_{2\alpha}$ and 8-*iso*-$PGF_{2\alpha}$, F_2-prostaglandins include isomers that are listed in Table S1. They all have the formula $C_{20}H_{34}O_5$, the molecular mass 354.48, and three hydroxyl groups at C9, C11 and C15. Two OH groups are positioned on the cyclopentane ring (C9 and C11 in 8-*iso*-$PGF_{2\alpha}$).

Table S1. Names and abbreviations of the F_2-prostaglandin investigated by Ferretti & Flanagan [4].

Abbr.	Prostaglandin F_2 isomer trivial name	IUPAC nomenclature
A	Prostaglandin $F_{2\alpha}$ ($PGF_{2\alpha}$)	(5*Z*,9*α*,11*α*,13*E*,15*S*)-9,11,15-Trihydroxyprosta-5,13-dien-1-acid
B	8-*iso*-Prostaglandin $F_{2\alpha}$ (8-*iso*-$PGF_{2\alpha}$)	8-*iso*-(5*Z*,9*α*,11*α*,13*E*,15*S*)-9,11,15-Trihydroxyprosta-5,13-dien-1-acid
C	15*R*-Prostaglandin $F_{2\alpha}$ (15*R*-$PGF_{2\alpha}$)	(5*Z*,9*α*,11*α*,13*E*,15*R*)-9,11,15-Trihydroxyprosta-5,13-dien-1-acid
D	Prostaglandin $F_{2\alpha}$ (9β,11α-$PGF_{2\alpha}$)	(5*Z*,9β,11*α*,13*E*,15*S*)-9,11,15-Trihydroxyprosta-5,13-dien-1-acid
E	Prostaglandin $F_{2\alpha}$ (9α,11β-$PGF_{2\alpha}$)	(5*Z*,9*α*,11b,13*E*,15*S*)-9,11,15-Trihydroxyprosta-5,13-dien-1-acid
F	8-*iso*-9β,11α-Prostaglandin $F_{2\alpha}$ (8-*iso*-9β,11α-$PGF_{2\alpha}$)	8-*iso*-(5*Z*,9b,11*α*,13*E*,15*S*)-9,11,15-Trihydroxyprosta-5,13-dien-1-acid
G	5-*trans*-Prostaglandin $F_{2\alpha}$ (5-*trans*-$PGF_{2\alpha}$)	(5*E*,9*α*,11*α*,13*E*,15*S*)-9,11,15-Trihydroxyprosta-5,13-dien-1-acid

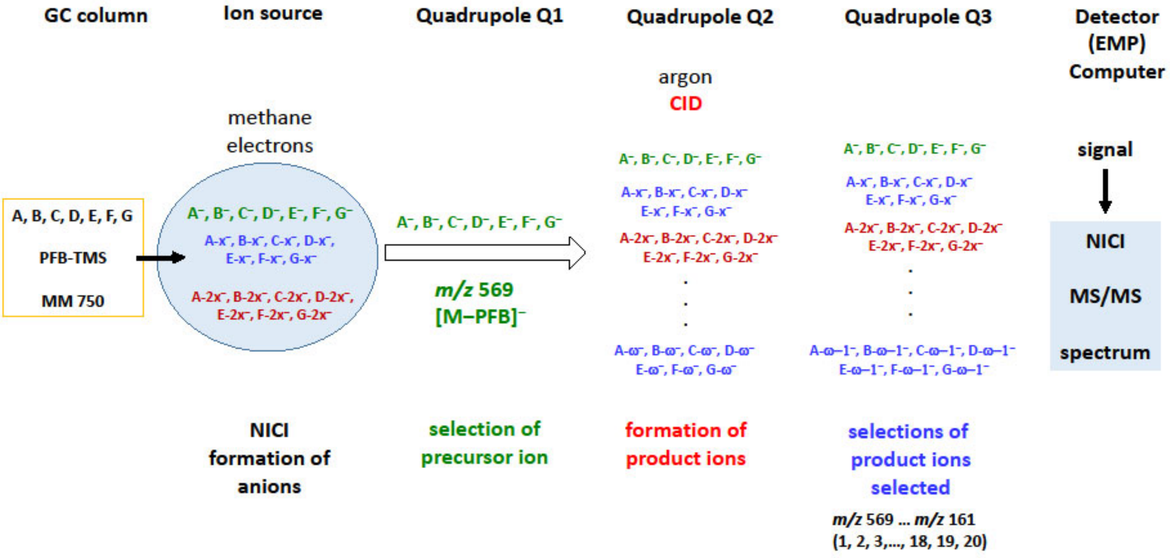

Figure S2. Simplified schematic of the GC-NICI-MS/MS analyses performed in individual experiments by Ferretti and Flanagan [4] on seven F_2-prostaglandins (A, B, C, D, E, F, G) as their PFB-TMS derivatives. NICI in the ion-source using methane as the reagent gas resulted in formation of several ions ($A\text{-}x^-$… $G\text{-}x^-$; $A\text{-}2x^-$… $G\text{-}2x^-$). The most abundant ions were *m*/*z* 569 due to $[M\text{−}PFB]^-$, i.e., A^-, B^-, C^-, D^-, E^-, F^-, and G^-; $^-$ symbolizes the negatively charged carboxylate group. These ions were selected by quadrupole Q1 and forwarded to the collision-chamber, the quadrupole Q2. Collision-induced dissociation (CID) of the precursor ions *m*/*z* 569 with argon atoms generated product ions due to elimination of TMSOH groups (90 Da) and CO_2 (44 Da) as neutral losses and decomposition of the backbone ($A\text{-}x^-$… $G\text{-}x^-$; $A\text{-}2x^-$… $G\text{-}2x^-$; $A\text{-}\omega^-$… $G\text{-}\omega^-$). The precursor ions and 19 product ions were selected by scanning the quadrupole Q3 ($A\text{-}x^-$… $G\text{-}x^-$; $A\text{-}2x^-$… $G\text{-}2x^-$; $A\text{-}\omega\text{–}1^-$… $G\text{-}\omega\text{–}1^-$) to generate the GC-NICI-MS/MS mass spectra. The collision energy was 32 eV for all F_2-prostaglandins [4].

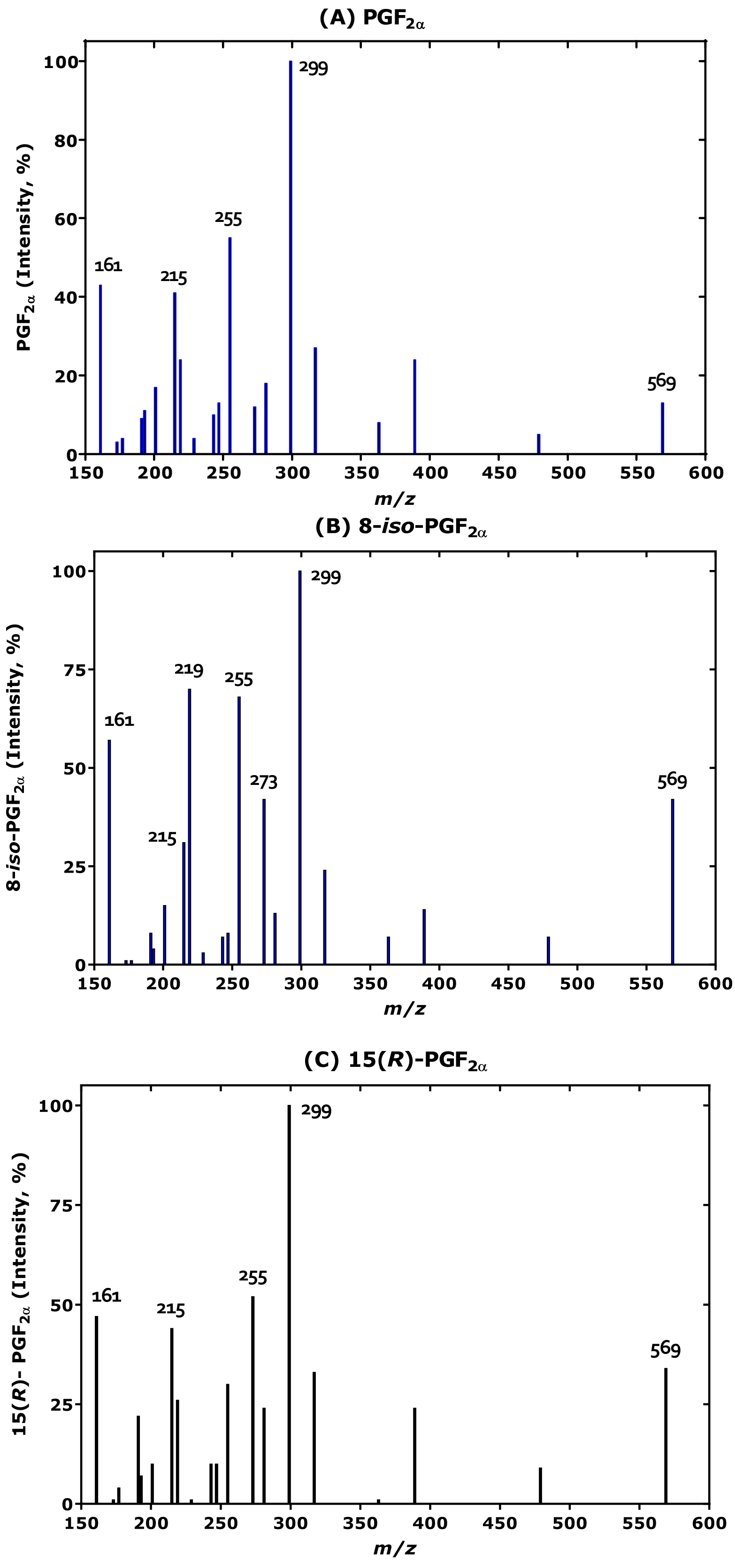
(A) PGF2α
PGF2α (Intensity, %)
m/z
161
215
255
299
569
(B) 8-iso-PGF2α
8-iso-PGF2α (Intensity, %)
m/z
161
215
219
255
273
299
569
(C) 15(R)-PGF2α
15(R)- PGF2α (Intensity, %)
m/z
161
215
255
299
569

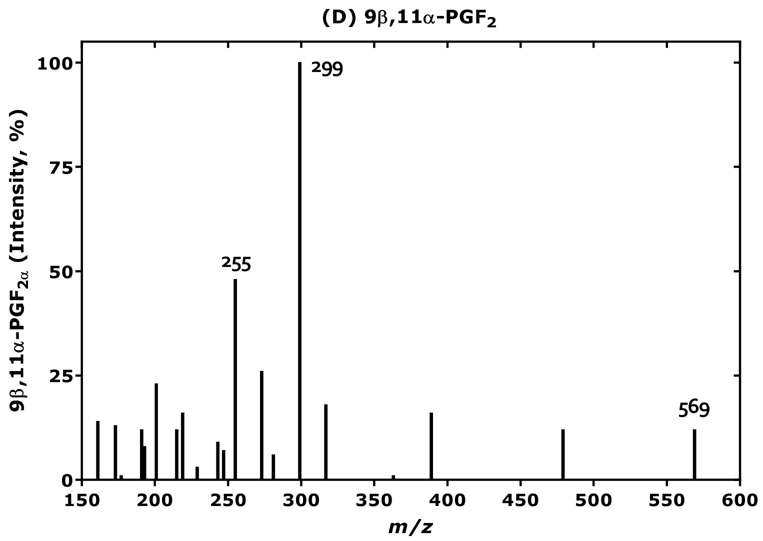
(D) 9β,11α-PGF2
9β,11α-PGF2α (Intensity, %)
100
75
50
25
0
299
255
569
150
200
250
300
350
400
450
500
550
600
m/z

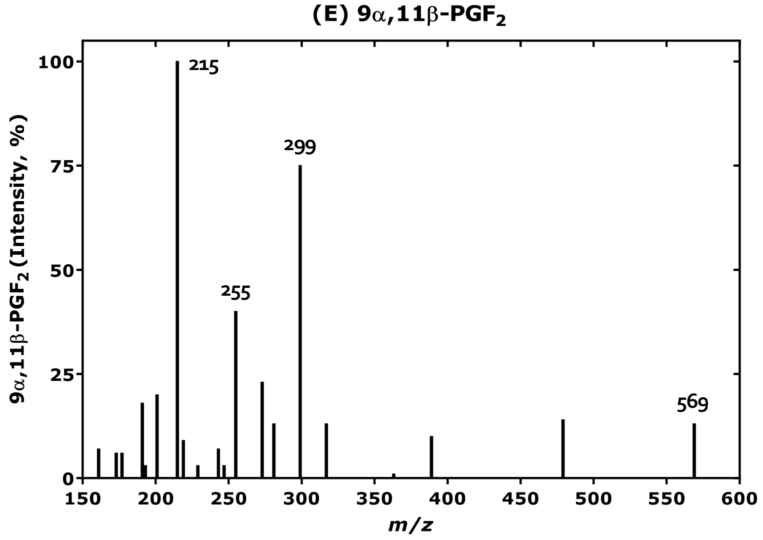
(E) 9α,11β-PGF2
9α,11β-PGF2 (Intensity, %)
100
75
50
25
0
215
299
255
569
150
200
250
300
350
400
450
500
550
600
m/z

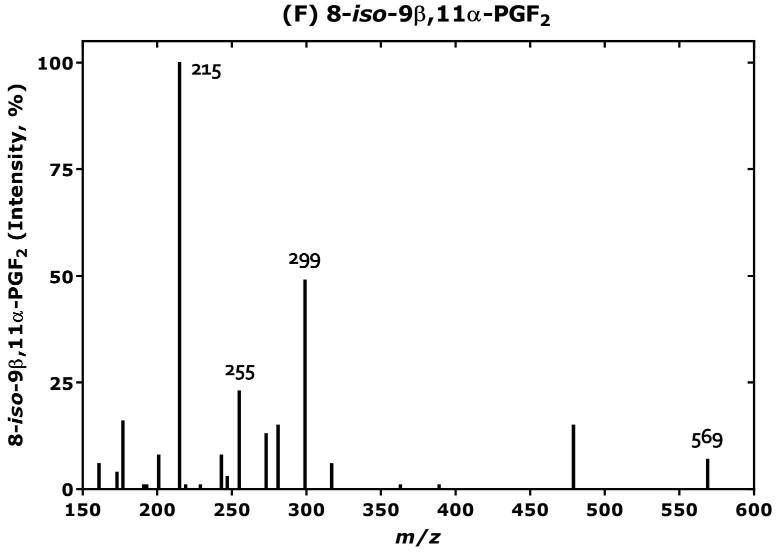
(F) 8-iso-9β,11α-PGF2
8-iso-9β,11α-PGF2 (Intensity, %)
100
75
50
25
0
215
299
255
569
150
200
250
300
350
400
450
500
550
600
m/z

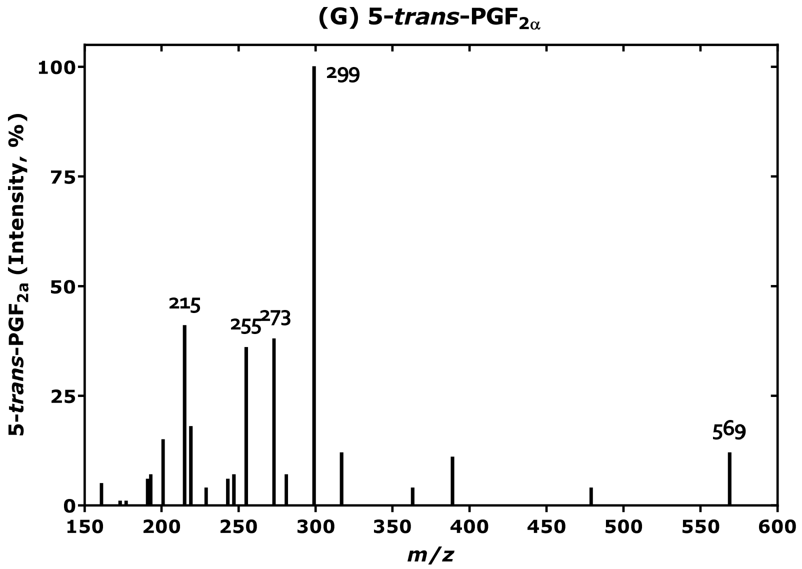

Figure S3. Product ions generated by CID of the precursor ions [M−PFB]⁻ with *m*/*z* 569 formed by NICI of the PFB-TMS derivatives of the prostaglandin F_2 isomers investigated in the study. The GC-NICI-MS/MS mass spectra were reconstructed by GraphPad Prism using the data reported by Ferretti and Flanagan [4].

ROC analyses of between the *m*/*z* values of the F2-prostaglnadins

Area under the ROC curve for PGF2a vs. PGF2a-isomer (mean ± SEM, n=20)

1) 8-iso-PGF2a vs.: AUC = 0.5100 ± 0.09459, *P* = 0.9138
2) 15(R)-PGF2a vs. PGF2a: AUC = 0.5213 ± 0.09423, *P* = 0.8181
3) 9b,11a-PGF2a vs. PGF2a: AUC = 0.5663 ± 0.09218, *P* = 0.4735
4) 9a,11b-PGF2a vs. PGF2a: AUC = 0.5913 ± 0.09095, *P* = 0.3235
5) 8-iso-9b,11a-PGF2a vs. PGF2a: AUC = 0.7075 ± 0.08340, *P* = 0.0248
6) 5-trans-PGF2 vs. PGF2a: AUC = 0.6363 ± 0.08932, *P* = 0.1404

ROC analysis of the data revealed AUC values for the ROC curves, which were statistically significantly different only for the comparison of $PGF_{2\alpha}$ with 8-*iso*-9β,11α-$PGF_{2\alpha}$: AUC = 0.7075 ± 0.0834, *P* = 0.0248 (mean ± SEM).

Correlation coefficients after Spearman from analyses between the *m*/*z* values of the F2-prostaglandins

Green-colored values are statistical significant, red-colored values are statistical not significant

	PGF2a	8-iso- PGF2a	15(R)- PGF2a	9b,11a-PGF2a	9a,11b-PGF2a	8-iso-9b,11a-PGF2a
8-iso- PGF2a	0,877					
15(R)- PGF2a	0,823	0,903				
9b,11a-PGF2a	0,637	0,740	0,674			
9a,11b-PGF2a	0,563	0,652	0,710	0,694		
8-iso-9b,11a-PGF2a	0,341	0,309	0,438	0,270	0,665	
5-trans-PGF2a	0,784	0,828	0,787	0,691	0,723	0,367

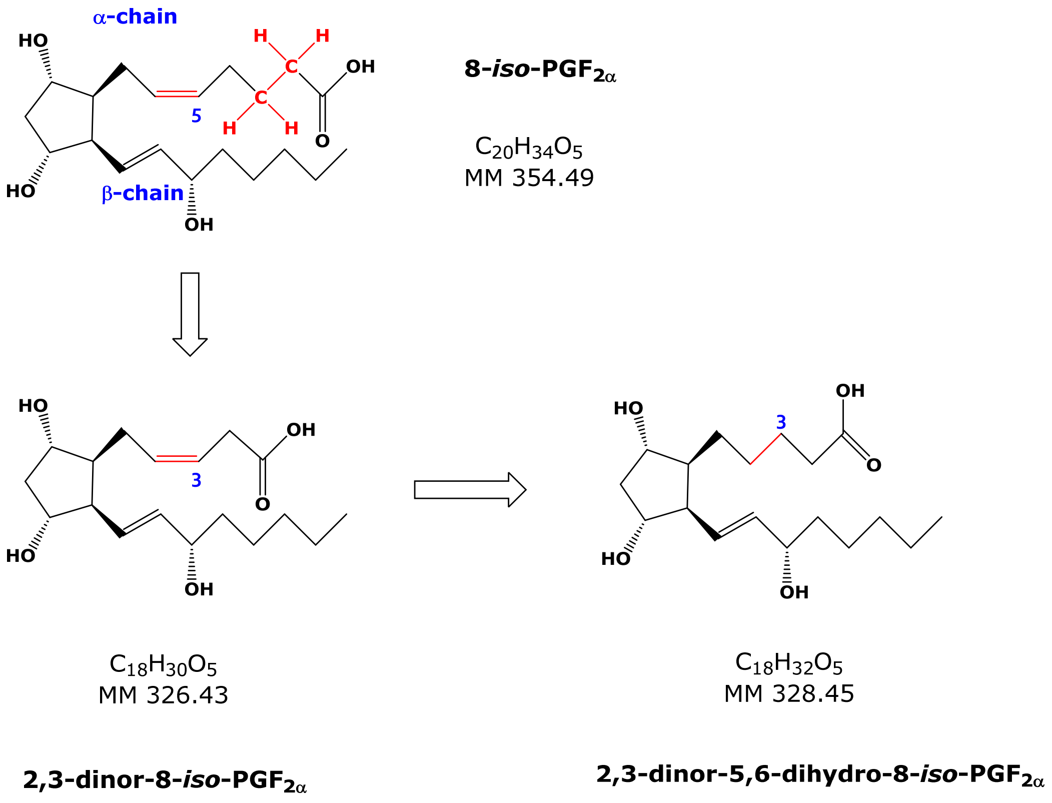

Figure S4. Chemical structures of 8-*iso*-$PGF_{2\alpha}$, 2,3-dinor-8-*iso*-$PGF_{2\alpha}$, and 2,3-dinor-5,6-dihydro-8-*iso*-$PGF_{2\alpha}$.
